# Exploratory rasch analysis of a static-99R clinical cohort assessment

**DOI:** 10.1371/journal.pone.0307216

**Published:** 2024-07-18

**Authors:** Christian Baudin, Anna Grimby-Ekman, Thomas Nilsson, Märta Wallinius, Peter Andiné

**Affiliations:** 1 Department of Psychiatry and Neurochemistry, Centre for Ethics, Law and Mental Health, Institute of Neuroscience and Physiology, Sahlgrenska Academy at the University of Gothenburg, Gothenburg, Sweden; 2 Department of Forensic Psychiatry, National Board of Forensic Medicine, Gothenburg, Sweden; 3 Biostatistics, School of Public Health and Community Medicine, Institute of Medicine, University of Gothenburg, Gothenburg, Sweden; 4 Forensic Psychiatric Clinic, Sahlgrenska University Hospital, Gothenburg, Sweden; 5 Research Department, Regional Forensic Psychiatric Clinic, Växjö, Sweden; 6 Department of Clinical Sciences Lund, Evidence-Based Forensic Psychiatry, Psychiatry, Lund University, Lund, Sweden; Nazarbayev University, KAZAKHSTAN

## Abstract

Modern test theory supplements the more prevalent classic methods for assessing test properties. However, such an assessment of the commonly used sexual recidivism risk assessment instrument, Static-99R, has yet to be attempted. This study evaluated the psychometric properties of said instrument using Rasch analysis. The clinical cohort assessed consisted of individuals with mental disorders convicted of a sexual offense (N = 146). Results showed that the original ten-item Static‑99R did not meet the Rasch model requirements, and revisions of the instrument with seven and nine items each only marginally improved performance. More reliable results could likely have been obtained with a larger, non-clinical sample and a more randomized distribution of missing data. Despite the consistently poor performance of item 3 (“Index non-sexual violence”) in all three analyses, reliability was slightly improved by dichotomizing the only two polytomous items in the Static-99R; items 1 (“Age at release from index offense”) and 5 (“Prior sexual offenses”). These results may be of interest considering the significant change of splitting the formerly dichotomous item 1 into four different response categories in the revision of Static-99 to Static-99R.

## Introduction

Actuarial risk assessment instruments are developed using empirically derived risk factors where the total score results in a probability of recidivism [1]. Arguably, Static-99R has become the de facto gold standard for actuarial assessments of recidivism risk in adults convicted of a sexual offense and is widely used by professionals and clinicians globally [2]. Its ten items are empirically linked to sexual recidivism, and very little training is required to obtain reliable results [3]. Interviewing the convicted person, while recommended, is not a prerequisite for carrying out an assessment. All items except items 1 and 5 are dichotomous, adding either 0 or 1 point to the total score, whereas item 1 awards -3, -1, 0, or 1 point, and item 5 either 0, 1, 2, or 3 points. The total score indicates which of five recommended risk levels is applicable. The risk level is in turn linked to certain probabilities for recidivism depending on the chosen norm group.

Both external and internal validity is important when validating an actuarial risk assessment instrument. External validity may refer to the instrument’s ability to discriminate high-risk individuals from low-risk individuals, whereas internal validity refers to how well the instrument is calibrated for a specific population. In order to accurately and proportionally allocate treatment resources according to models like the risk-need-responsivity model, both discrimination and calibration are essential when validating risk assessment instruments [4, 5]. Discrimination is the statistical concept of accurately separating individuals demonstrating high risk from individuals demonstrating low risk. It is commonly examined using the area under the curve (AUC) derived from receiver operating characteristics (ROC) and odds ratios (OR) from logistic regression models [6–8]. Calibration pertains to absolute recidivism risk, or how great the probability is that an individual demonstrating high risk will re-offend and, conversely, how small the probability is that an individual demonstrating low risk will not. When examining how well-calibrated an instrument is, it has been suggested that the E/O index (and its derivative, the P/E index) and the Brier score be used [7, 9, 10].

Another important aspect of instrument performance that is often overlooked is construct validity. This aspect concerns how well an instrument captures the entire spectrum of the underlying trait (or construct) it aims to measure. A common approach is classical test theory (CTT), typically using Cronbach’s alpha coefficient, factor analysis, and correlation analysis [11]. Classical test theory is supplemented by modern test theory (MTT), where data is fitted to a logistic regression model for repeated measures [12, 13]. One method within modern test theory is Rasch analysis [14], which is part of the Item Response Theory (IRT) family of statistical methods. While originally developed for quantifying problem-solving ability in the school environment, Rasch analysis has also been applied to several scales used in clinical psychiatry [15–21] and forensic contexts [22–24]. The construct validity of the Static-99 and its revised version, the Static-99R, has been examined in previous studies using factor analysis [25, 26] and a non-parametric IRT procedure [27] on non-clinical samples. These studies have investigated the latent trait structure and its correlation with the theoretical constructs of the instrument. However, while IRT attempts to create a model that reflects the data, Rasch analysis tests the instrument’s metric measurement properties by evaluating whether the data fit an expected model [28], making it a valuable tool for developing, adapting, and evaluating the psychometric properties of scales and similar instruments.

While Static-99R has been found to predict recidivism well in many different populations using methods for measuring internal and external validity, to our knowledge, its construct validity has not yet been examined using modern test theory when estimating the recidivism risk of convicted individuals with mental disorders. This is of particular importance due to the diverse characteristics within this population, including varying clinical diagnoses, recidivism rates, victim preference, modus operandi, level of sexual arousal, personal and criminal histories, demographics, age, and more [29–40]. Therefore, this explorative study aims to assess and evaluate the psychometric properties of Static-99R by using risk assessments of a Swedish clinical cohort of individuals with mental disorders who have been convicted of sexual offenses. The evaluation involves two stages: first, by testing the Rasch model requirements using the above data, and second, by identifying and describing any discrepancies in the model.

## Methods

### Legal setting

In Sweden, convicted persons suffering from major mental illnesses such as psychotic disorders, severe personality disorders, and severe developmental disorders are generally precluded from receiving prison sentences. Instead, these convicted persons are sentenced to open-ended compulsory forensic psychiatric care [41]. The National Board of Forensic Medicine commonly aids the court’s decision by undertaking a four-week pretrial forensic psychiatric investigation (FPI).

### Study cohort, data collection, and data handling

Included in this study were adult males who, between January 1, 1993, and December 31, 1997, underwent a court-ordered FPI in Sweden and were subsequently convicted for a sexual offense against an adult [42]. Most of the cohort (62.3%, n = 91) were born in Sweden, whereas roughly one in five originated from other Nordic countries or other parts of Europe (8.2%, *n* = 12, and 8.9%, *n* = 13, respectively). The rest (20.5%, *n* = 30) were of non-European descent. All 146 individuals had been assessed using the Static-99R by three of the authors in a previous study [43]. The authors had extensive clinical experience with various risk assessment instruments. In addition, the corresponding author participated in an online course held by a certified Static-99R trainer in the USA and, in turn, instructed the other two assessors. The collective assessments of the cohort produced an ICC of .89 (CI 95% .76-.94, p < .001), commonly interpreted as between “good” and “excellent” [44] or well above “strong” [45]. The ten Static-99R items are listed in order according to the coding rules [3]: 1. “Age at release from index sexual offense”; 2. “Ever lived with a lover”; 3. “Index non-sexual violence–Any convictions”; 4. “Prior non-sexual violence–Any convictions”; 5. “Prior sexual offenses” [charges and convictions counted separately]; 6. “Four or more prior sentencing dates (excluding index)”; 7. “Any convictions for non-contact sexual offenses”; 8. “Any unrelated victims”; 9. “Any stranger victims”; and 10. “Any male victims”.

Actual release dates were unavailable for 29 persons, affecting one of the items of the Static-99R, but missing data is permissible in Rasch modeling. Offense data was collected from the National Council for Crime Prevention’s convictions register, and the FPI reports from the National Board of Forensic Medicine. For additional details regarding the scoring and validity of the Static-99R assessments in this cohort, please see Baudin et al. [43].

### Rasch modeling and statistical analysis

Rasch modeling is commonly used to evaluate and interpret the psychometric properties of scales and questionnaires in health, education, and psychological assessments attempting to measure unidimensional constructs or traits. Developed by Danish mathematician Georg Rasch, the method supplements classical test theory [46, 47] and is available for dichotomous and polytomous items [48–51]. The Rasch model assumes that a participant’s responses to several items estimate his or her ability and item difficulty using a logistic regression model for repeated measures [12]. If the data fits the Rasch model, the scale or instrument may be assigned qualities like other well-defined measures, such as length or weight, where a single unit is identical to any other part of the scale. The method avoids floor and ceiling effects and transforms an instrument comprising ordinal scale items into an interval scale [51]. The instrument’s psychometric properties are revealed by measuring how and to what degree the study cohort assessment data differs from a hypothetical, perfect model, which, in turn, allows for theoretical improvements to the scale [52]. In short, Rasch modeling tests whether or not—and how well—an instrument metrically measures an underlying trait and offers insight into said psychometric properties of the scale. When conducting explorative studies on subgroups of larger populations, such as the present study, few response categories and samples between 150 and 250 subjects are suggested [52].

Rasch analysis is an iterative process performed using several sequential steps. Listed below are the steps generally recommended for a study using the method, although not necessarily in the following order:

- Presenting the person separation index (PSI), a test reliability measure. The PSI utilizes the same formula as Cronbach’s alpha, and the two measurements are comparable, but PSI uses the score logits instead of the raw score itself [44, 50, 53].
- Testing the general model fit, consisting primarily of the item fit and person fit values. Model fit is examined by a) analyzing the z-score transformed fit residuals for all items and all persons and b) analyzing how much the item-person interaction fit residuals deviate from the expected standard deviation (*SD*) of 1 [12, 50]. There are no rigid criteria for the fit residuals, but values between 0.1 and 0.7 are frequent [54]. A standard recommendation is removing items or persons demonstrating a fit residual of less than -2.5 or more than +2.5 [11, 12, 55, 56]. In addition to the fit residuals, the item-trait interaction chi-square probabilities reflect the degree of invariance of item difficulty across the measured trait [12]. A statistically significant interaction indicates that the difficulty of an item may differ for two persons presenting contrasting levels of the underlying trait, which is unwanted.
- Graphically illustrating how well-targeted the items are to the sample’s persons using a person-item threshold map. The mean person logit value should be centered around 0 and cover the same region as the logits of the items [12, 50, 56].
- Examining the response category threshold ordering of polytomous items. A response category awarding a lower score should indicate a lower level of the trait, whereas a response category awarding a higher score should indicate a higher level of the trait. Any categories in between the two extremes should follow a gradual increase in both score and trait level. If not, one usually collapses the number of response categories by rescoring the affected item(s) until threshold order is achieved [12, 50, 55].
- Assessing the presence and potential effect of differential item functioning (DIF). DIF is a form of item bias that may occur when subgroups within a sample respond differently to an item despite presenting equal levels of the trait. DIF is identified using a two-way variance analysis (ANOVA) of the standard residuals comparing observed and expected values. Statistically significant findings indicate a potential DIF [11, 55, 56]. Uniform DIF indicates the presence of consistent differences between the groups tested, which can be corrected by splitting the persons and testing the affected items separately for each subgroup. In contrast, non-uniform DIF indicates inconsistent differences across the measured trait and generally necessitates the removal of the item from the scale altogether [12].
- Checking for any local dependency of items by looking at unidimensionality and response dependency [57]. Unidimensionality (a single underlying trait) is tested using a principal component analysis (PCA). The PCA analyzes item residuals of the discrepancy between observed and expected responses for meaningful patterns. Items are separated into two groups depending on the item residual loadings (+0.3 and less than -0.3) [58]. The two groups are then compared using a series of *t-*tests, and if 5% or fewer of the *t-*tests are statistically significant, the scale is generally deemed unidimensional [58]. Response dependency occurs when a response to one item may affect the response to another item. It is identified by examining a residual correlation matrix [50]. Generally, interactions between two items with a value of +0.3 or higher are considered problematic [56, 57]. These tests do not definitively ascertain whether or not there is more than one underlying trait, but the results may offer insight to the researcher [58].

This study conducted three Rasch analyses. The first analysis evaluated how well the original ten-item Static-99R met the Rasch model requirements. The second and third analyses examined two modified versions, each addressing and resolving the disordered thresholds of items 1 and 5 differently. In the second analysis, the disordered thresholds were resolved by maximizing the PSI, while in the third analysis, overlapping categories were collapsed based on clinical judgment. The latter changes aimed to enhance the results by making clinically relevant adjustments to the items and response categories rather than focusing solely on any one statistical improvement.

The following dichotomous (yes/no) clinical, social, and demographic variables were tested for DIF: psychotic disorder, intellectual disability, substance use disorder (SUD), Nordic background, secondary school diploma, and having a partner. Sex and age are usually tested when performing Rasch analyses, but since Static-99R may be applied to males only [3] and contains one item involving the person’s age, these variables were excluded.

A Bonferroni corrected *p*-value of 0.05 divided by the number of significance tests conducted for a given part of the Rasch analysis was consistently used. As with any estimate of a type I error, the recommended alpha varies, but a Bonferroni correction on an alpha of either 0.01 or 0.05 is typical for Rasch analyses [11, 12, 50, 56]. Here, an alpha of 0.05 was deemed reasonable, given the exploratory nature of this study.

Concerning the use of statistical software, RUMM 2030+ (RUMM Laboratory Pty Ltd, Perth, WA) was used for all Rasch analyses, while descriptive statistics unrelated to Rasch were conducted using jamovi 1.2.22 (The jamovi project, Sydney, NSW).

### Ethics statement

The authors take full responsibility for the data’s integrity and accuracy and have made every effort to avoid inflating statistically significant results. This study was approved by the Regional Ethical Review Board at the University of Gothenburg (377–17, T1056-17). Study participants were not considered directly affected by the registry-based research at the time of the original data collection, resulting in no informed consent being collected. These decisions were considered appropriate by the ethical review boards in 1997 and 2017. Static-99R scores used in the analyses were extracted from the assessment protocols on October 26, 2020. While the original FPI reports contained information that could identify individual participants, this information was neither needed nor accessed in writing this manuscript.

## Results

### Rasch analysis of the original ten-item version of Static-99R

**Fig 1:**
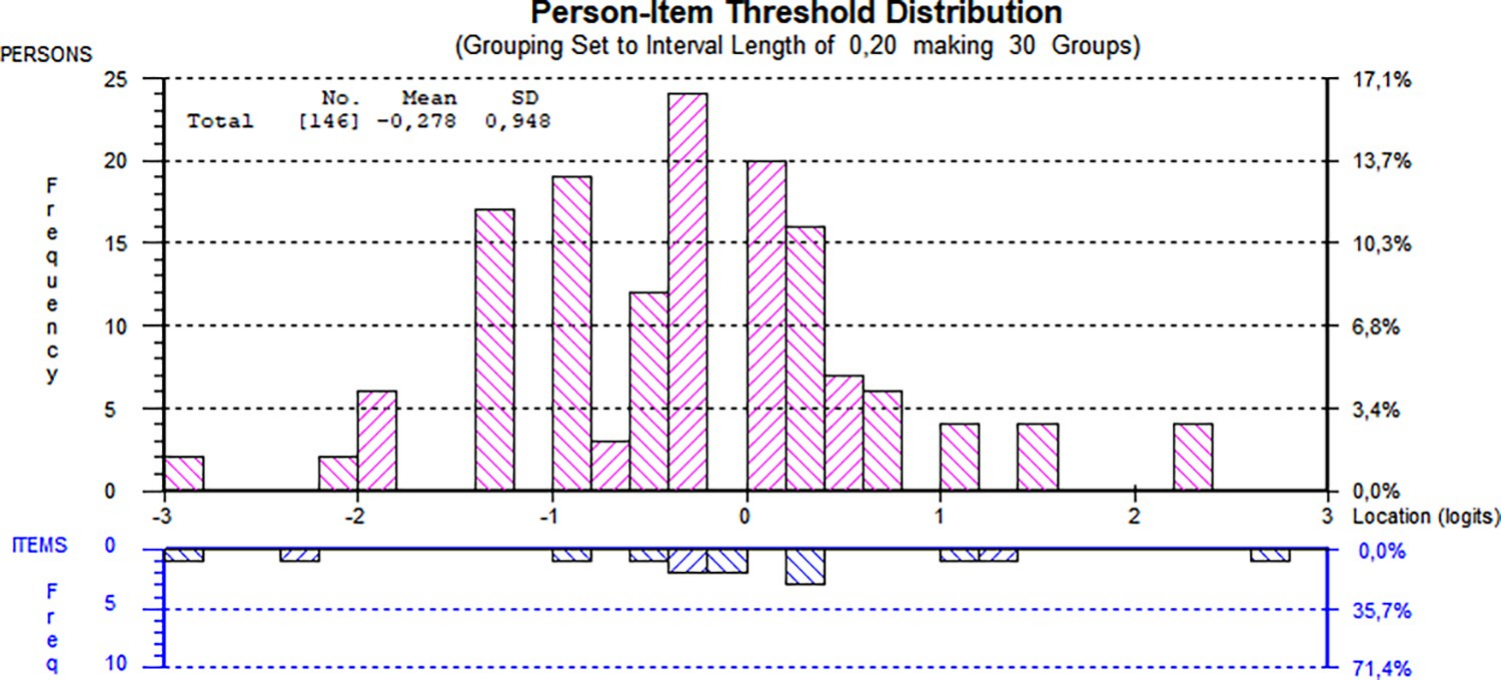
Person-item threshold distribution using all items. All 146 individuals assessed using the original ten items of the Static-99R risk assessment instrument were included. Be aware that figures generated by the software use a decimal comma instead of a decimal point.

Considering that Static-99R consists of both dichotomous and polytomous items, a likelihood-ratio test could not be conducted [51]. As such, the partial credit model was used. The original ten items of Static-99R demonstrated an overall PSI of 0.53 with no extreme persons discovered, indicating a low but reasonable power of analysis of fit for an exploratory study. The mean person location was -0.28, suggesting reasonable targeting of the items, with only a slightly visible skewness of person frequency compared to the item threshold spread seen in Fig 1. The negative value indicates that the cohort demonstrated a lower level of the underlying trait measured by Static-99R, while Fig 1 shows adequate person-item spread by having both persons and items located throughout the entirety of the logit x-axis.

The item-trait interaction chi-square was statistically significant (χ^2^ = 75.4, *df* = 20, *p* < .001), suggesting that general item difficulty inconsistently quantified the measured trait. This is further supported by the fit residual standard deviation exceeding the expected value of 1 (*SD* = 1.67). These two indications can primarily be explained by item 3, demonstrating a problematic fit residual of 3.60, well outside of +/- 2.5, considered a critical value [11]. Regarding person fit, while the standard deviation of person fit residuals indicated a slight misfit (*SD* = 0.58 compared to the expected 1), no individual fit residual higher than +2.5 or lower than -2.5 was observed (range = -1.45 to 1.91).

**Fig 2:**
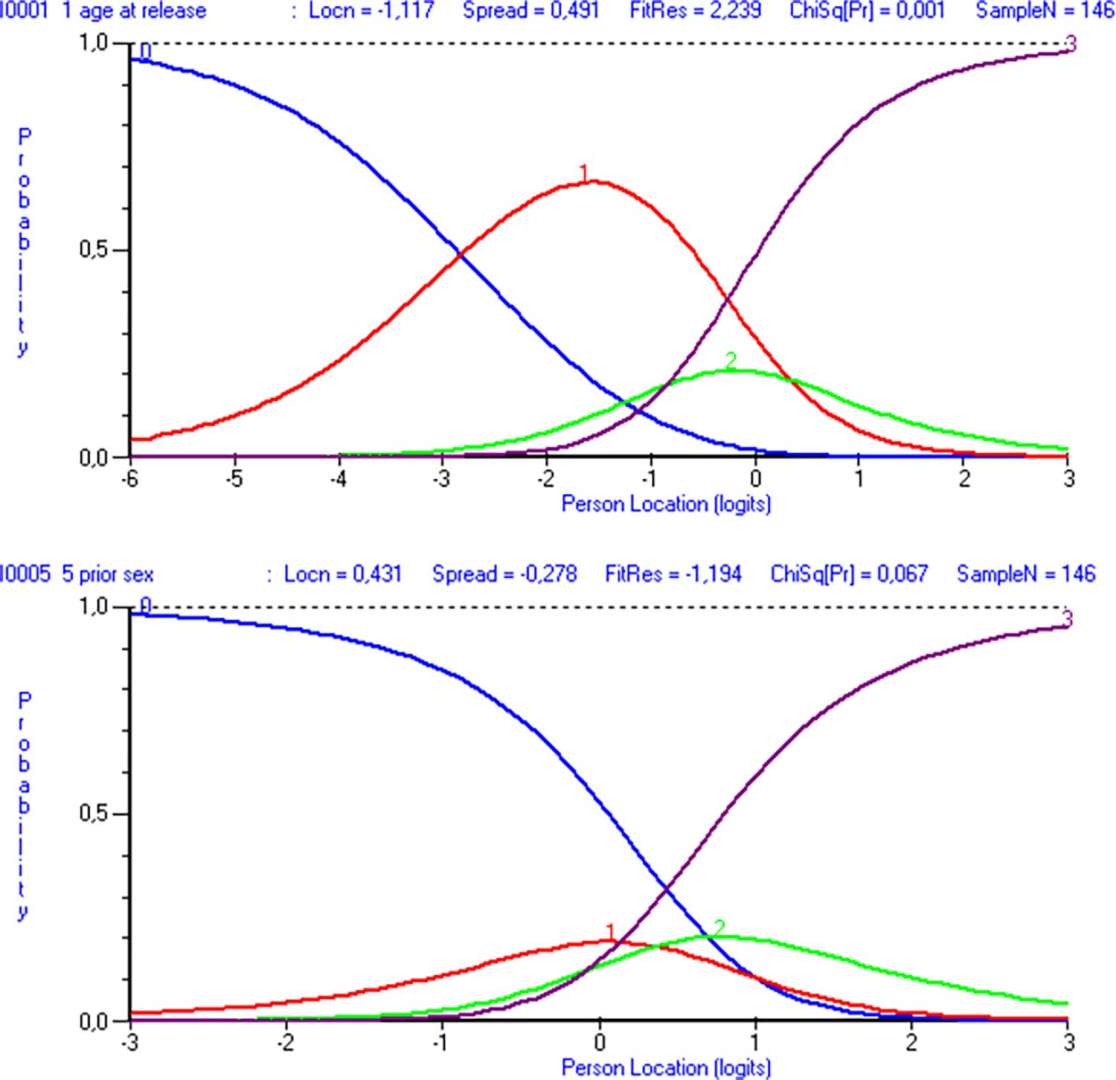
Category probability curves for items 1 and 5 of the Static-99R. Both item 1 (“age at release from index offense”) and item 5 ([number of] “prior sexual offenses”) present four response categories each (N = 146). Be aware that figures generated by the software use a decimal comma instead of a decimal point.

The two polytomous items alone demonstrated disordered thresholds: items 1 and 5, with four response categories each. This is visualized in Fig 2 by both items failing to form monotonic regions barely overlapping across the x-axis. Instead, response category 1 for item 1 and response categories 1 and 2 for item 5 are wholly eclipsed by the others.

Out of the ten items and six clinical, social, and demographic groups tested for DIF group interactions, three items demonstrated uniform DIF (*p* < .001) for three separate groups: item 1 and substance use disorder, item 3 and intellectual disability, and item 6 and secondary school diploma.

Two tests were performed for local independence. Firstly, by examining the residual pattern, two potential subscales were detected: one comprising item 1 and item 3 and one comprising items 5 through 9. Using all persons, since no extremes were detected, the PCA found merely 0.7% of the *t*-tests to be significant. This is well below the cut-off of 5%, generally suggesting that the requirement of unidimensionality was met. Secondly, regarding response dependency, two item pairs demonstrated an interaction slightly above the critical value of 0.3: items 4 and 6 (0.327) and items 8 and 9 (0.368). In other words, persons affirming one item in a pair tended to affirm the other in the same pair and vice versa.

Overall, the Rasch analysis of Static-99R exhibited several psychometric concerns related to construct validity for the ten items when assessing a clinical cohort convicted of a sexual offense. In order to study this further, two alternate sets of Rasch analyses were conducted. The first set adhered to the strict requirements of the Rasch model and is presented in the following section. The second set aimed to meet the Rasch model’s main requirements while also considering a slightly more clinical focus.

### Rasch analysis of a revised seven-item version of Static-99R with strict Rasch requirements focus

It is advisable to make changes in the Rasch analysis one at a time because any modifications can have complex effects on subsequent analyses, including rescoring items, removing items, resolving DIF, or changing the model frame of reference [11, 59]. The sequential steps for the revised seven-item version of Static-99R are outlined in Table 1.

**Table 1:** First sequential analyses.

Step	Change	PSI	Item-trait interaction	Mean person location	Item misfit	Threshold ordering
1	Collapsing response categories for item 1	0.55	χ^2^ = 72.7, *df* = 20, *p =* .001	-0.26	Item 3 (3.76)	Item 1 (0,1,2,3 -> 0,1,1,2)
2	Collapsing response categories for item 5	0.54	χ^2^ = 61.1, *df* = 20, *p =* .001	-0.28	Item 3 (3.80)	Item 5 (0,1,2,3 -> 0,1,1,2)
3	Removal of item 3	0.59 (0.58, 2)	χ^2^ = 66.2, *df* = 18, *p =* .001	-0.35 (-0.31, 2)	Item 1 (2.55)	None
4	Removal of item 1	0.59 (0.47, 13)	χ^2^ = 46.1, *df* = 16, *p* < .001	-0.57 (-0.32, 13)	Item 2 (3.20)	“
5	Removal of item 2	0.60 (0.44, 18)	χ^2^ = 23.2, *df* = 14, *p =* .06	-0.57 (-0.31, 18)	None	“
6	DIF analysis	“	“	“	“	“
7	Test for local dependency	“	“	“	“	“

Sequential analyses of the original ten-item Static-99R risk assessment instrument resulting in seven items adhering to the Rasch requirements (N = 146). Please note that PSI and mean person location are presented for both the total cohort and, in parentheses, for when extreme persons are excluded, followed by the number of excluded persons. Threshold ordering describes numbered response categories before the collapse and -> after the collapse.

**Fig 3:**
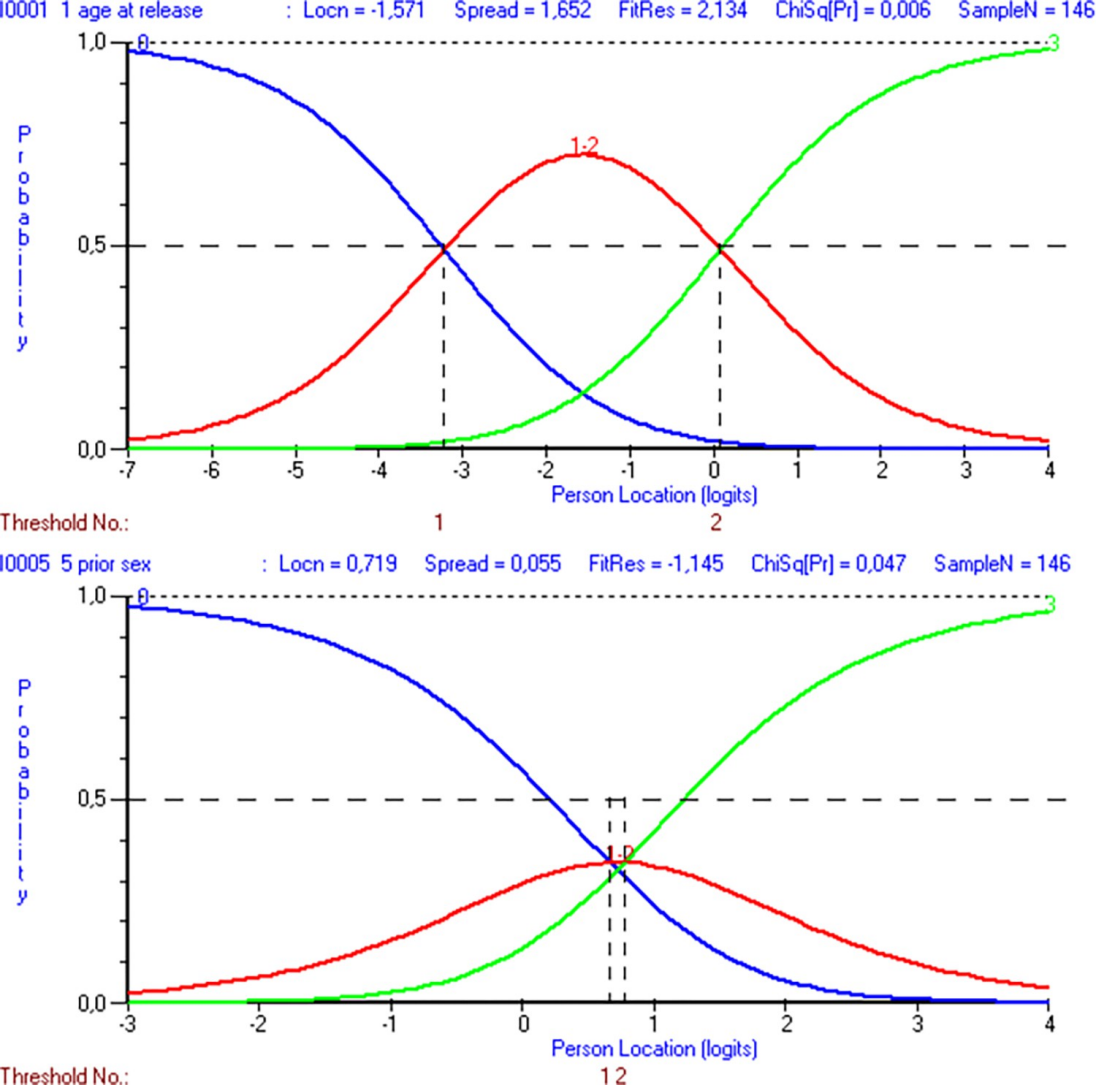
Category probability curves for modified items 1 and 5 of the Static-99R. Both item 1 (“age at release from index offense”) and item 5 ([number of] “prior sexual offenses”) demonstrate ordered thresholds with three response categories each (N = 146). For item 1, category 1 (“aged 35 to 39.9”) and category 2 (“aged 40 to 59.9”) were combined, and for item 5, category 1 (“charges 1, 2” or “convictions 1”) and category 2 (“charges 3–5” or “convictions 2, 3”) were combined.

Due to disordered thresholds in polytomous items 1 and 5 in the original analysis, it was preferable to collapse the response categories. This was done by exploring various ways of collapsing item 1, then item 5, and then combinations of both, measuring success by maximizing the PSI and achieving ordered thresholds. For a visualization of these steps, please see Fig 3. These adjustments led to a slightly improved scale reliability and item-trait interaction, as demonstrated in Table 1, step 2.

As noted in the original analysis, only item 3 demonstrated a problematic fit residual, necessitating its elimination from the scale. Removing the item caused a ripple effect that led to two more items showing fit residuals that warranted their removal: first, item 1, then item 2. Removing all three items again led to better scale reliability and item-trait interaction (Table 1, step 5) but resulted in 18 extreme persons.

No clinical, social, or demographic groups demonstrated any statistically significant interactions when analyzing the seven-item scale for DIF. This revised version underwent a PCA, but no clear indication of multidimensionality (3.42% of the *t*-tests were significant), and no response dependencies were found in the residual correlation matrix. Therefore, the revised version was considered locally independent with higher reliability than the ten-item version and a statistically non-significant item-trait interaction chi-square, which is preferable to a significant one in this type of analysis.

**Fig 4:**
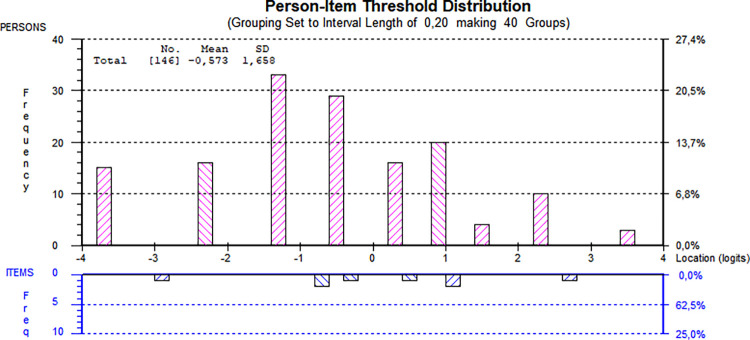
Person-item threshold distribution using seven items. All 146 individuals assessed by the revised seven-item Static-99R risk assessment instrument. Be aware that figures generated by the software use a decimal comma instead of a decimal point.

In contrast to the original ten-item version, the mean person location deviated further from zero. However, as shown in Fig 4, while the person-item spread was similar to the ten-item version, the seven items no longer encompassed the full range of the person locations. Consequently, the range was narrower, resulting in lower precision for individuals with locations at the extremes of the scale.

### Rasch analysis of a revised nine-item version of Static-99R with a more pragmatic focus

While the seven-item version met the Rasch requirements to the best extent possible, given the available data, it resulted in losing three of the ten items, only slightly improved internal consistency (PSI), and unfortunately led to increased mistargeting. Therefore, a nine-item revision was tested as a clinically focused and pragmatic version of Static-99R that aimed to follow the Rasch requirements closely.

**Fig 5:**
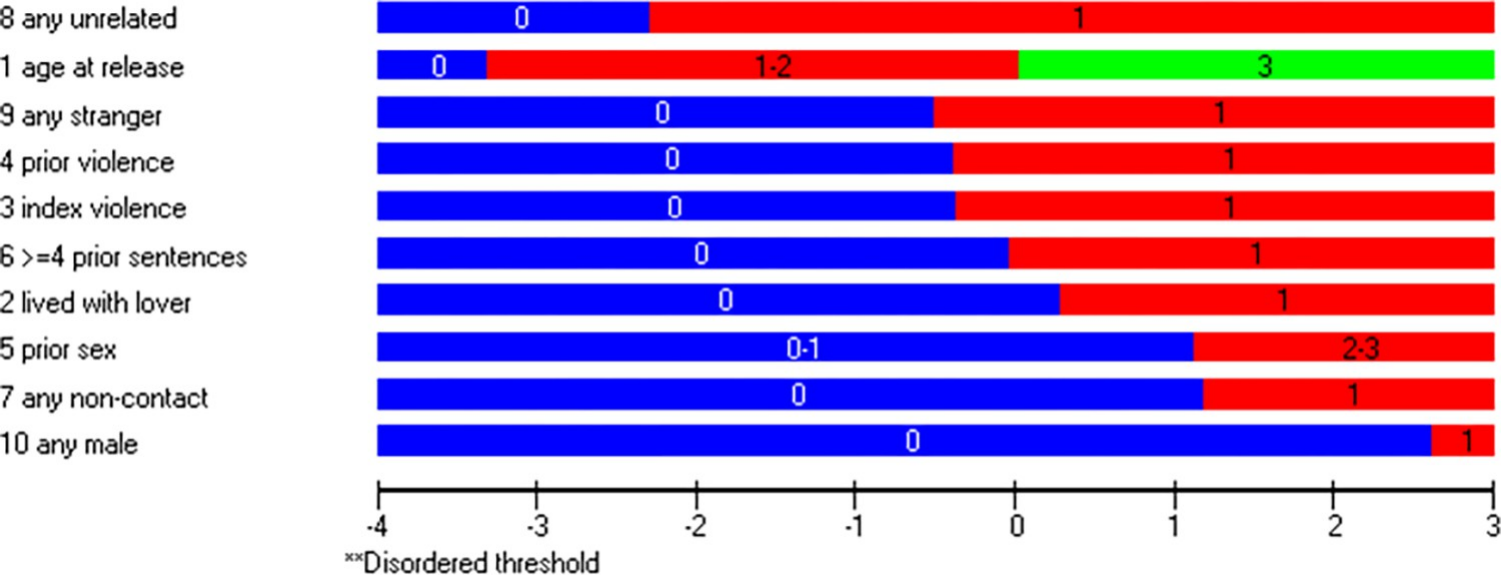
Threshold map during step 2 of the alternative sequential analyses. All 146 individuals assessed using all ten items of the Static-99R risk assessment instrument, ordered by ascending item location in logits on the x-axis. Please note that at this analysis stage, the disordered thresholds for polytomous items 1 and 5 have been corrected, but the ill-fitting item 3 has not yet been removed.

In this nine-item version, item 1 was collapsed as described above, but with item 5, a different approach was taken. Item 5 was split into two categories, as shown in Table 2, step 2, in contrast to the three shown in Fig 3. This change resulted in a slight decrease in reliability but an increased item-trait interaction chi-square. All items were still present in the analysis at this point. As shown in Fig 5, the item difficulty spread for all ten items visualized by the threshold map demonstrated a high difficulty for item 10 and a low difficulty for item 8. However, item 3 still demonstrated a problematic fit residual and was removed (Table 2, step 3). After removing item 3 (not depicted in any figure), reliability and targeting were slightly improved, whereas item-trait interaction was not. No further items had problematic fit residuals, although item 1 was just below the critical value (2.45).

**Table 2:** Alternative sequential analyses.

Step	Change	PSI	Item-trait interaction	Mean person location	Item misfit or DIF	Threshold ordering
1	Collapsing response categories for item 1	0.55	χ^2^ = 72.7, *df* = 20, *p =* .001	-0.26	Item 3 (3.76)	Item 1 (0,1,2,3 -> 0,1,1,2)
2	Collapsing response categories for item 5	0.52	χ^2^ = 59.7, *df* = 20, *p* < .001	-0.32	Item 3 (3.31)	Item 5 (0,1,2,3 -> 0,0,1,1)
3	Removal of item 3	0.57 (0.56, 2)	χ^2^ = 60.1, *df* = 18, *p* < .001	-0.39 (-0.35, 2)	None	None
4	Test for local dependency	“	“	“	“	“
5	DIF analysis	“	“	“	Item 1 (SUD), uniform	“
6	DIF resolve	0.60 (0.59, 2)	χ^2^ = 52.3, *df* = 20, *p* < .001	-0.23 (-0.19, 2)	None	“

Sequential analyses of the original ten-item Static-99R risk assessment instrument resulting in nine items more closely adhering to the Rasch requirements (N = 146). Please note that PSI and mean person location are presented for both the total cohort and, in parentheses, for when extreme persons are excluded, followed by the number of excluded persons. Threshold ordering describes numbered response categories before the collapse and -> after the collapse.

**Fig 6:**
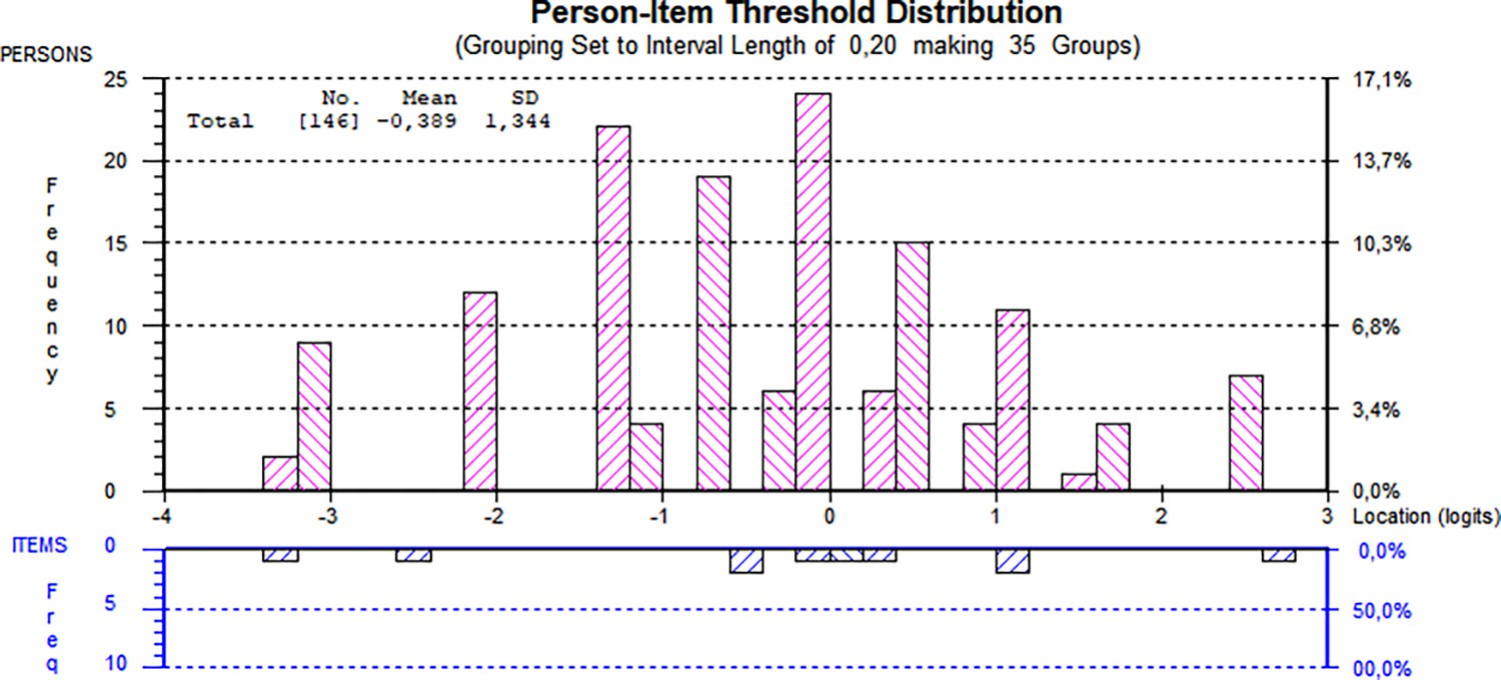
Person-item threshold distribution using nine items. All 146 individuals assessed by the revised nine-item Static-99R risk assessment instrument. Be aware that figures generated by the software use a decimal comma instead of a decimal point.

Neither multidimensionality nor response dependency was observed, with only 1.37% of the PCA *t*-tests yielding significant results. Fig 6 illustrates that the nine items exhibited a comparable distribution of scores among individuals and items as the original version, without any drawbacks seen in the seven-item version.

**Fig 7:**
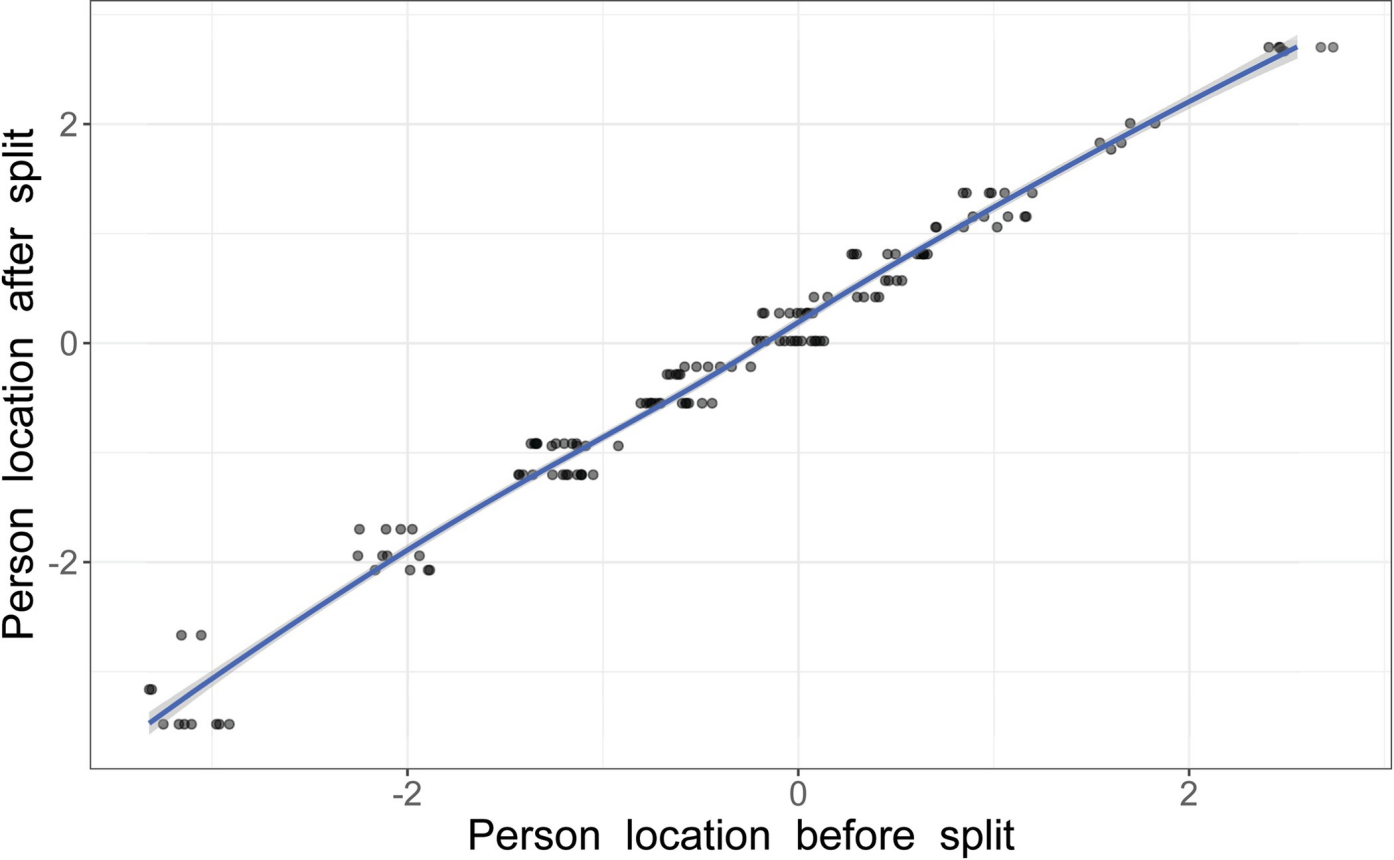
Person location scatterplot. The scatterplot visualizes person location in logits before and after splitting item 1 according to affiliation with the “substance use disorder yes/no” group of the 146 individuals assessed by the revised nine-item Static-99R risk assessment instrument. Only minor differences were found, with a slope of 1.048 (CI 95% 1.03–1.07, Pearson’s *r* = 0.994, *r*^*2*^ = 0.988).

When testing for DIF, item 1 demonstrated a statistically significant group-factor interaction, indicating a potential uniform DIF concerning substance use disorder. To address this issue while maintaining item structure, the affected item was split into two groups based on group affiliation: one for individuals with substance use disorder and one for those without. [59]. When resolved, the difference between the two groups was confirmed visually by the differences in the ICCs and mathematically by distinct item location shifts: -0.67 for those with a substance use disorder and -2.44 for those without. The resolved sub-items and all other items still demonstrated adequate model fit with no problematic fit residuals. While this outcome was anticipated, it is not always guaranteed [59]. In addition, the Fig 7 scatterplot graphically presents the changes in person location before and after the DIF split.

The resolved nine-item version displayed a higher PSI than during previous steps while maintaining a similar quality to the seven-item version. The item-trait interaction chi-square showed improvement, remaining statistically significant. Additionally, targeting was further improved whether or not the two extreme persons were included.

### Limitations

Firstly, it is preferred to use a large, nationwide, non-clinical sample to properly utilize the advantages of Rasch analysis when evaluating the psychometric properties of a scale. Unfortunately, such a sample is currently unavailable in Sweden, whereas data was available concerning the smaller cohort in the present study. As such, collecting and archiving Static-99R results for research purposes is recommended going forward—including original tally sheets to ensure all total and individual item scores are retained. This type of data collection may reach beyond common practice, and depending on jurisdiction, relevant regulatory bodies need to be engaged in order to take advantage of modern test theory properly.

As is common in studies involving the assessment of an instrument, missing data affected some of the analytic steps. Rasch analysis effectively handles missing data but assumes that the missing data is random within the dataset [56]. Although less than 2% of the total responses were missing, this exclusively affected item 1, resulting in a loss of one in five responses for that item. The missing data may explain why item 1 showed more extensive issues than the other items in Static-99R. Additionally, this made it impossible to conduct new tests for local dependency after splitting the item for DIF in the nine-item version of the instrument.

## Discussion

This paper has presented a modern test theory-based psychometric analysis of Static-99R using Rasch modeling, which, to our knowledge, has not been previously attempted. Three versions of Static-99R were analyzed: 1) an unadulterated version of the ten original items, 2) a revised seven-item version that adhered closely to Rasch requirements, and 3) a revised nine-item version that was guided by the Rasch model while still attempting to maintain clinical relevance. By presenting these three analyses, the paper compares Static 99R’s psychometric performance, allowing readers to understand the differences between the original instrument and two simplified, hypothetical versions.

In all three versions of Static-99R, psychometric issues arose to some degree. Firstly, and most importantly, reliability never exceeded a PSI of 0.60—well below the preferred value of 0.70 [50]. Instruments with fewer items tend to demonstrate lower PSI values [11, 60, 61], although this is not always the case [11, 62]. While PSI is not enough to gauge a scale’s performance, it is an important measure that affects and is affected by many parts of the Rasch analysis iterative process [11]. Therefore, the findings should be interpreted with a higher degree of caution than is ideal and should in no way be overstated. Secondly, poor item-trait interactions showed that item difficulty may vary among individuals with different levels of the underlying trait, which is undesirable for any scale. Items 1 and 2 were the main contributors to this effect, but it was resolved in the seven-item version of the instrument, where both items were excluded. Thirdly, both polytomous items 1 and 5 displayed disordered thresholds, indicating too many response categories, considering the clinical nature of Static-99R [12]. All analyses considered, none of the Static-99R versions presented in this study were suitable for transforming logit person estimates into a metric score for parametric analysis, which is a common objective of using Rasch analysis.

It is worth noting that targeting was successful overall, with persons presenting a slightly lower level of the underlying trait on average. Effective targeting is essential in clinical settings, as considerable mistargeting may lead to reduced overall scale reliability and the inability of the scale to differentiate individuals based on their trait level [11, 12, 56]. Additionally, all three versions of the instrument appeared to meet the requirements of unidimensionality, as shown in the PCA tests. There were no signs of unintentional subscales within the instrument, supporting the assumption that Static-99R measures only one specific trait. This finding contrasts with two previous studies [25, 26] and might be a false positive related to the abovementioned weak PSI or, possibly, an actual difference between the populations studied. In addition to the potentially positive results regarding unidimensionality, the person-item spread was satisfactory in all three versions, as seen in Figs 1, 4 and 6. This indicated that the cohort generally consisted of individuals displaying a wide range of trait levels and that, in general, the items adequately measured that trait.

On closer examination of the three separate versions of Static-99R, the original ten-item version demonstrated specific psychometric issues, some of which were resolved in the seven- and nine-item versions. Items 1, 3, and 6 demonstrated DIF group interactions with three distinct groups: individuals with substance use disorder, intellectual disability, and those with secondary school diplomas. These interactions were uniform, meaning the likelihood of responding in a certain way was consistent across the groups. The interactions revealed that individuals with substance use disorder tended to be older at release from their index offense, those with intellectual disability were less likely to be sentenced for a non-sexual violent offense as part of their index offense, and individuals with a secondary school diploma had fewer prior sentencing dates. This suggests that individuals with substance use disorders may have more extended periods of incapacitation, either due to longer prison sentences or by being placed in open-ended compulsory forensic psychiatric care. Scoring this item can be difficult, partly because the age of the convicted person at release is not always readily available and partly because what constitutes release may be challenging to determine when various jurisdictions have differing definitions of what constitutes release. Naturally, the optimal context would be a risk assessment performed as part of a release procedure in Canada or the USA, where age at release is apparent, and the definition of release perfectly matches that of the Static-99R manual. The DIF group interaction related to intellectual disability was inconclusive due to the rarity of the diagnosis (13 individuals) and a lack of group interactions with non-sexual violent offenses prior to the index offenses (item 4). Given the association of education with prosocial behavior, the link between having secondary school diplomas (one-third of the cohort) and fewer prior convictions was expected. While failing to complete secondary school is relatively uncommon in Sweden, of those later committing partner violence, an alarmingly high proportion lack a secondary school diploma [63]. Although the item-group interactions were statistically evident, the psychometric issue would have been more significant if all affected items had been linked to a single group, which was not the case.

Local dependency was only observed in two item pairs: items 4 and 6 and items 8 and 9. The former item pair relates to the number of previous convictions, both sexual and non-sexual, and the latter to the relationship between the victim and the convicted. Given these circumstances, the response dependency is unsurprising.

The revised seven-item version demonstrated the best psychometric results among the three tested versions, although the difference was slight. The seven-item version was the only one that did not exhibit item-trait invariance, as three underperforming items were eliminated (items 1, 2, and 3). Additionally, this version had the largest number of extreme persons (18 total).

Finally, the revised nine-item version performed better than the original ten-item set but still displayed some issues related to disordered thresholds, item-trait interaction, and DIF. As with the original ten-item version, item 1 exhibited DIF group interactions with the substance use disorder group, although the effect was small but statistically significant. As a result, an attempt was made to split the item based on group affiliation. This splitting slightly impacted model fit and targeting but made further analyses of local dependency impossible due to complications with missing data. However, by visually comparing person location in logits before and after the DIF split using a scatterplot, the differences between the two groups were found to be minimal and primarily affected individuals on the lower end of the trait spectrum, as illustrated in Fig 7.

In summary, all three versions showed minor but noteworthy differences in performance. While targeting was not a prominent issue in the present explorative study, a non-clinical cohort might have demonstrated better targeting than the current one. Given the assumed differences between a forensic psychiatric population and the typical prison population (also known as “routine sample”) for which the Static-99R was originally designed, this is particularly important. Previous studies have shown similar distinctions between clinical and non-clinical samples when performing Rasch analyses on other scales and instruments [56, 62].

The threshold map of the nine-item version of Static-99R, prior to the removal of item 3, as shown in Fig 5, may be of particular interest as it illustrates the distribution of trait difficulty across the items after accounting for threshold disorder, which did not markedly differ among the three versions. The difficulty appears relatively evenly spread across all dichotomous items and the single polytomous item left in the revised version. Item 10, when affirmed, indicated a noticeably higher risk of being located at the higher end of the trait spectrum. In other words, a person in the cohort who had previously sexually victimized a male was more likely to receive a notably high total score on Static-99R despite the item only contributing one incremental point to the total score. This pattern is consistent with research suggesting that individuals who target males are more likely to be reconvicted for new offenses [64].

### Conclusions

While none of the three versions of the Static-99R showed a satisfying model fit using Rasch analysis, certain findings may still interest both clinicians and researchers, as long as the implications are not exaggerated. Specifically, reliability was improved by collapsing items 1 and 5 into dichotomous variables, limiting the number of response categories for the assessor. The finding is notable, considering the original dichotomous item 1 was split into four different response categories in the revision of Static-99 to Static-99R. However, more reliable results could likely have been obtained with a larger, non-clinical sample and a more randomized distribution of missing data.

## Supporting information

S1 Data Static-99R scores and DIF variables, anonymized. (XLSX)
